# Dynamic Phosphorylation of NudC by Aurora B in Cytokinesis

**DOI:** 10.1371/journal.pone.0153455

**Published:** 2016-04-13

**Authors:** Kimberly N. Weiderhold, Maria Fadri-Moskwik, Jing Pan, Michiya Nishino, Carol Chuang, Arpaporn Deeraksa, Sue-Hwa Lin, Li-Yuan Yu-Lee

**Affiliations:** 1 Program in Integrative Molecular and Biomedical Sciences, Baylor College of Medicine, Houston, Texas, United States of America; 2 Department of Medicine, Section of Allergy Immunology and Rheumatology, Baylor College of Medicine, Houston, Texas, United States of America; 3 Department of Molecular and Cellular Biology, Baylor College of Medicine, Houston, Texas, United States of America; 4 Department of Translational Molecular Pathology, The University of Texas M.D. Anderson Cancer Center, Houston, Texas, United States of America; Institut de Génétique et Développement de Rennes, FRANCE

## Abstract

Nuclear distribution protein C (NudC) is a mitotic regulator that plays a role in cytokinesis. However, how NudC is regulated during cytokinesis remains unclear. Here, we show that NudC is phosphorylated by Aurora B, a kinase critical for cell abscission. NudC is co-localized with Aurora B at the midbody and co-immunoprecipitated with Aurora B in mitosis. Inhibition of Aurora B by ZM447439 reduced NudC phosphorylation, suggesting that NudC is an Aurora B substrate *in vivo*. We identified T40 on NudC as an Aurora B phosphorylation site. NudC depletion resulted in cytokinesis failure with a dramatic elongation of the intercellular bridge between daughter cells, sustained Aurora B activity at the midbody, and reduced cell abscission. These cytokinetic defects can be rescued by the ectopic expression of wild-type NudC. Reconstitution with T40A phospho-defective NudC was found to rescue the cytokinesis defect. In contrast, reconstitution with the T40D phospho-mimetic NudC was inefficient in supporting the completion of cytokinesis. These results suggest that that dynamic phosphorylation of NudC by Aurora B regulates cytokinesis.

## Introduction

Cytokinesis is the final stage of cell division. Cell abscission is a critical step in this process, without which the daughter cells may fuse back together to form a single cell with an aberrant amount of DNA or aneuploidy. Many proteins and protein complexes are known to be involved in cytokinesis [1–5]. However, the process and regulation of cell abscission remains to be fully understood.

One protein that plays a critical role in cytokinesis and cell abscission is the mitotic kinase Aurora B, the catalytic component of the chromosomal passenger protein complex (CPC) [5–8]. Aurora B regulates many steps throughout mitosis, including DNA condensation during prophase, kinetochore-microtubule attachment during prometaphase and metaphase, cleavage furrow formation during anaphase and telophase, and cell abscission to complete cell division [7,9–11]. Aurora B has been shown to participate in these activities by switching binding partners and substrates between early versus late mitosis [7,8,12,13]. By understanding Aurora B binding partners and substrates during cytokinesis and abscission, a more detailed role of Aurora B in regulating cytokinesis can be elucidated.

Nuclear distribution protein C (NudC) is a 42-kDa protein that is highly conserved from fungus to man [14–19]. NudC was originally identified in the filamentous fungus *Aspergillus nidulans* in a screen to identify temperature-sensitive *nud* mutants defective in the migration of nuclei into the fungal hyphae [14]. All the *nud* genes encode components or regulators of the dynein/dynactin motor complex [20]. As a dynein-associated protein, NudC is localized to the Golgi [21], microtubule organizing center [21–24] and cell cortex [22], and plays an evolutionarily conserved role in dynein-dependent functions, ranging from transport of intracellular cargo [20,23] to the migration of neurons during neocortical brain development [22,25–27].

We identified mammalian NudC in a screen to identify genes upregulated in response to mitogenic stimuli [18], suggesting that NudC may play a role in cell division. Elevated levels of *NudC* mRNA and protein were found in tissues and cells with high proliferative potential. These include multiple tissues and cell types in the developing *C*. *elegans* [16] and amphibian [17] embryos, and mammalian hematopoietic cells [28,29], megakaryocytes [30], T lymphoma cells [18], neuroblastoma cells [21], and prostate cancer cells [31]. During cell division, NudC is localized to the centrosomes, kinetochores, mitotic spindle, central spindle, and midbody matrix [32–35]. In mammalian cells, knockdown of NudC results in mitotic defects including misattachment of microtubules to kinetochores during prometaphase [32,33], chromosome congression errors in metaphase [32,33], and an inability to complete cytokinesis [34,35]. Thus, NudC also plays a critical role in regulating mitotic progression. To perform these diverse functions, NudC is likely differentially regulated at various stages of mitosis and cytokinesis. In this study, we identified NudC as a new substrate of Aurora B and examined whether Aurora B plays a role in regulating NudC functions during cytokinesis.

## Materials and Methods

### Antibodies and Inhibitors

The following antibodies were used for immunoprecipitation (IP) and immunoblotting (IB, dilutions shown): NudC (70/1, rabbit, 1:1000) [32], NudC (G1, goat, 1:1000) [33], NudC (2D9, mouse, 1:2000) [32], Aurora B (BD, mouse, 1:800), Aurora B (Abcam, rabbit, 1:2000), α-tubulin (GeneTex, rabbit, 1:2000), α-tubulin (Sigma, mouse, 1:1000), β-tubulin(tub2.1) (Sigma, mouse, 1:1000), and FLAG (Sigma, mouse, 1 μl/mg protein for IP). The following antibodies were used for immunofluorescence (1:1000, unless otherwise indicated): NudC (G1, goat) [33], pSerNudC (R2, rabbit) [32], Borealin (MBL International, mouse), PRC1 (Abcam, rabbit), MKLP-1 (Cell Signaling, rabbit), and pTSS-INCENP^834-902^ (gift of Dr. Michael Lampson, University of Pennsylvania; rabbit) [36], Spc25 (gift of Dr. P. Todd Stukenberg, University of Virginia Medical Center; rabbit; 1:700) [37], and CREST-SH autoserum (gift of Dr. Bill R. Brinkley, Baylor College of Medicine; human; 1:10,000) [38]. Protease inhibitors and nocodazole, the microtubule depolymerizing agent, were purchased from Sigma. ZM447439, an Aurora B inhibitor, was purchased from Tocaris and used at a final concentration of 2 μM.

### Cell Culture and Synchronization

HeLa cells were obtained from the Tissue Culture Core facility in the Department of Molecular and Cellular Biology at Baylor College of Medicine and are free of mycoplasma. HeLa cells were cultured in DMEM (Invitrogen) supplemented with 10% fetal bovine serum (FBS) (Atlanta Biologicals). Cells were synchronized using two protocols. In the first method, HeLa cells were incubated with 100 ng/ml nocodazole, a microtubule depolymerizing agent, for 16 h to enrich for cells in a prometaphase-like phase. Asynchronously growing (Asy) (no treatment) and nocodazole-synchronized mitotic (M) cells were harvested with a cell scraper or by a mitotic shake-off. In the second method, HeLa cells were treated with 2 mM thymidine for 15 h to block cells at the G1/S transition, released for 10.5 h to allow cells to enter into the next G1 phase, and then treated with a second thymidine block for 13.5 h. Cells were then released for 5.5 h and then incubated for 3 h in either 20 ng/ml nocodazole to enrich for early mitotic cells (P, for prometaphase and metaphase) or 12 ng/ml nocodazole to allow efficient release of the nocodazole-arrested cells to enter into the later stages of mitosis (A, for anaphase, telophase and cytokinesis), and harvested. Cell pellets were lysed in ice-cold RIPA buffer (150 mM NaCl, 20 mM Tris pH 8, 1.5 mM EDTA, 5 mM EGTA, 0.1% Triton X-100, 5% glycerol) supplemented with 1 mM PMSF, mammalian protease-inhibitor cocktail (Sigma), 5 mM Na_3_VO_4_, 5mM NaF, and serine-threonine and tyrosine phosphatase inhibitor cocktails (Sigma).

### Immunoprecipitation

Protein concentrations were determined by a Bradford assay (Bio-Rad). Total cell lysates were pre-cleared with protein G sepharose beads (GE) for 1 h at 4°C, combined with fresh protein G sepharose beads and primary antibody, and then rotated head over tail overnight at 4°C. Beads were pelleted, washed 4 times in RIPA buffer, and boiled in LDS Sample Buffer (Invitrogen) with 5% β-mercaptoethanol for 5 min at 95°C. Eluted proteins were resolved by SDS-PAGE and transferred to a nitrocellulose membrane. Membranes were immunoblotted with anti-Aurora B antibody (mAb 1:800) overnight, followed by anti-mouse HRP-coupled secondary antibody for 1 h at room temperature, and developed by enhanced chemiluminescence as suggested by the manufacturer (Thermo Scientific).

### RNA Interference

The small interfering RNA (siRNA) for NudC (5’–AACACCTTCTTCAGCTTCCTT– 3’ NM-006600, nucleotides 204–224) (Dharmacon) was described previously [32–34]. Firefly (Photonius pyralis) luciferase siRNA was used as a control (Dharmacon). In initial experiments, cells were also co-transfected with siGLO RISC-free siRNA (Dharmacon), a fluorescent non-targeting control oligonucleotide, at a 10:1 siRNA:siGLO ratio, to mark siRNA uptake in transfected cells. HeLa cells (3 x 10^4^ cells) were plated onto poly-L-lysine coated 18-mm coverslips (Fisher Scientific) in a 12-well dish for immunofluorescence, using antibiotic-free OptiMEM (Invitrogen) supplemented with 10% FBS for 24 h. Appropriate siRNA (120 pmol) was diluted in 24 μl pure OptiMEM. In a separate tube, 6 μl Oligofectamine (Invitrogen) was diluted in 100 μl pure OptiMEM, incubated at room temperature for 5 min, added to the diluted siRNA mixture, and allowed to incubate for 20 min at room temperature. The siRNA mixture was then added to the cells and incubated for 72 h to ensure a good knockdown of NudC.

### Transient DNA Transfection

HeLa cells were cultured in antibiotic-free DMEM supplemented with 10% FBS to a density of 70–80% confluency in 12 well (or 10 cm) culture dishes and transfected using Lipofectamine 2000 (Invitrogen). Briefly, 0.7 μg (or 8–10 μg) of the appropriate plasmid DNA was diluted in 100 μl (or 1.5 ml) of pure OptiMEM. In a separate tube, 1.75 μl (or 25 μl) of Lipofectamine were diluted in 100 μl (or 1.5 ml) pure OptiMEM and incubated for 5 min at room temperature. The DNA and Lipofectamine mixtures were combined, incubated at room temperature for 20 min, and added to the cells to incubate at 37°C for 24 h.

### GST-NudC Fusion Proteins

The PCR primers for generating the Glutathione-S-transferase (GST)-human NudC truncation series are shown in Table 1. Glutathione-S-transferase (GST) fusion protein cDNA constructs were transformed into E. coli strain BL-21. Bacteria were grown in LB media until the OD_600_ reached between 0.6 and 0.8 that corresponded to mid-log phase, induced with 0.1 mM isopropyl-beta-D-thiogalactopyranoside (IPTG) for 3 h at room temperature, pelleted, lysed in ice-cold phosphate buffered saline (PBS) supplemented with a bacterial protease inhibitor, phosphatase inhibitors and PMSF, sonicated, and rocked at 4°C for 15 min with 1% Triton X-100. Bacterial lysates were clarified and protein concentration was determined using a BSA standard. Glutathione-Sepharose 4B beads (GE Healthcare) were added to the clarified supernatant and proteins were allowed to bind to the beads with rocking for overnight at 4°C.

**Table 1:** PCR primers for GST-human NudC truncation constructs.

Forward Primers	hNudC Constructs	Reverse Primers
5’-GGC*ggatcc*ACC**ATG**GGCGGAGAGCAG-3’	**N1** hNudC (1–49 aa)	5’-CAG*gcggccgc*CCCCTTCTTCTCCTC-3’
5’-GGC*ggatcc*ACC**ATG**GGCGGAGAGCAG-3’	**N2** hNudC (1–143 aa)	5’-CAG*gcggccgc*CCTCCTTCCCTGG-3’
5’-GGC*ggatcc*ACC**ATG**GGCGGAGAGCAG-3’	**N3** hNudC (1–212 aa)	5’-CAG*gcggccgc*CCCTTGAGCCCCAC-3’
5’-GGC*ggatcc*ACC**ATG**GGCGGAGAGCAG-3’	**N4** hNudC (1–276 aa)	5’-CAG*gcggccgc*CTTCGTGTTGATCTCAGG-3’
5’-GGC*ggatcc*ACC**ATG**GGCGGAGAGCAG-3’	**FL** hNudC (1–331 aa)	5’-CAG*gcggccgc*CCGTTGAATTTAGCCTTGG-3’
5’-CAG*ggatcc*ACC**ATG**GCAGAGAAGCTTATCACAC-3’	**C4** hNudC (50–331 aa)	5’-CAG*gcggccgc*CCGTTGAATTTAGCCTTGG-3’
5’-CAG*ggatcc*ACC**ATG**GGGCCCCAGATC-3’	**C3** hNudC (101–331 aa)	5’-CAG*gcggccgc*CCGTTGAATTTAGCCTTGG-3’
5’-CAG*ggatcc*ACC**ATG**GATACTGAGGAAGATGAGG-3’	**C2** hNudC (144–331 aa)	5’-CAG*gcggccgc*CCGTTGAATTTAGCCTTGG-3’
5’- CAG*ggatcc*ACC**ATG**GACGGCAAGGTGG- 3’	**C1** hNudC (237–331 aa)	5’-CAG*gcggccgc*CCGTTGAATTTAGCCTTGG-3’

Note, BamHI (*ggatcc*) and NotI (*gcggccgc*) were used in the Forward and Reverse primers, respectively.

### GST Pulldown Assay

HeLa cells were synchronized as described above into asynchronously cycling (Asy), P (early prometaphase and metaphase) or A (anaphase, telophase and cytokinesis) population, and lysed in ice-cold RIPA buffer as described above. Cell lysates (400 μg at 1 mg/ml) were pre-cleared with Glutathione-Sepharose beads for 1 h at 4°C and incubated with either 20 μg of GST alone or GST-NudC fusion protein for overnight at 4°C with head over tail rotation. The beads were washed 3 times in RIPA buffer and then boiled in LDS sample buffer, resolved by SDS-PAGE and transferred to a nitrocellulose membrane. Membranes were blotted for anti-Aurora B antibody followed by anti-mouse HRP-coupled secondary antibody as described above.

### *In vitro* IP Kinase Assay

HeLa cells were co-transfected with 3 μg FLAG-tagged wild type or kinase dead (K109R) Aurora B construct and 7 μg Myc-tagged INCENP construct for 24 h. Cells were lysed in Kinase Lysis Buffer (20 mM Tris, pH 7.5, 1% NP-40, 250 mM NaCl, 2 mM EGTA, 1 mM EDTA) supplemented with both protease and phosphatase inhibitors. Aurora B was immunoprecipitated using anti-FLAG antibody (4 μg/ml lysates). *In vitro* IP kinase assays were performed as previously described [39]. Briefly, each kinase reaction contained 5 μCi 32P-γ-ATP (25 Ci/mmol; MP Biomedicals) in Kinase Reaction Buffer (20 mM Tris, pH 7.5, 1 mM EGTA, 10 mM MgCl2, 50 μM ATP, protease and phosphatase inhibitors). Substrates were used in equimolar concentrations, including 4.8 μg of GST-NudC proteins, 1 μg histone H3 (Sigma) as a positive control and 1μg GST alone as a negative control. Aurora B was added last to the reactions to prevent substrate-mediated kinase inhibition [40], and the reactions were incubated at 30°C for 30 min with agitation every 10 min. Reaction products were resolved by SDS-PAGE, and the gel was stained with Coomassie brilliant blue, dried down and developed by autoradiography. In some experiments, labeled proteins were transferred to a nitrocellulose membrane, developed by autoradiography, and further analyzed by immunoblotting for Aurora B. In samples using ZM447439 to inhibit Aurora B activity, 2 μM ZM447439 was added to all of the buffers during Aurora B immunoprecipitation and kinase reaction.

### Immunofluorescence Imaging

HeLa cells plated on poly-L-lysine coated coverslips in 12-well plates were rinsed with 37°C PHEM buffer (60 mM 1,4-Piperazinediethanesulfonic acid dipotassium salt [K-PIPES], 25 mM HEPES [pH 6.9], 10 mM EGTA, and 4 mM MgSO4) (all from Sigma) and then fixed in fresh 4% paraformaldehyde (Electron Microscopy Sciences) in PHEM buffer for 20 min at room temperature. The coverslips were then washed with ice-cold PBS, extracted with 0.5%Triton X-100 diluted in cold PHEM buffer, incubated for 20 min at room temperature, blocked overnight at 4°C in antibody solution (0.1 M PIPES, 1 mM MgSO4, 1mM EGTA, 1.83% L-lysine, 1% BSA, 0.1% NaN3, pH 7.2 with KOH and 2% milk), washed 2 times in cold PBS, and incubated with the primary antibody diluted in antibody solution overnight at 4°C. Following primary antibody incubation, the coverslips were washed 3 times in cold PBS, incubated with the secondary antibody diluted in antibody solution for 3 h at 4°C, washed 3 times in cold PBS, and mounted using ProLong Gold antifade reagent with DAPI (Invitrogen). Slides were visualized on a Nikon TE2000 widefield microscope system (Nikon, Lewisville, TX), and images were acquired using a Photometrics Cool Snap ES camera, analyzed using NIS-Elements AR 3.2 software (Nikon), and presented using Adobe PhotoShop CS (Adobe Systems Inc.).

### Quantification of Aurora B at Kinetochores in siNudC Cells

For measuring the immunofluorescence intensity of Aurora B at kinetochores, HeLa cells were transfected with siLuc or siNudC for 72 h, and cells undergoing unperturbed mitosis were fixed and stained with the CREST autoserum to mark the inner centromere region and either Aurora B or Spc25. Widefield images were acquired as z-stacks with a step size of 0.3 μm using a 100X oil/1.45 NA objective. Identical exposure times were used between siLuc and siNudC samples. Images were deconvolved using AutoDeblur/AutoVisualize software (Media Cybernetics, Silver Spring, MD), and maximum projections of deconvolved images were made. Intensity measurements were made essentially as described [41]. Briefly, individual kinetochores were identified by CREST staining and marked by a region of interest (ROI). The average intensity of signals in both the CREST channel and the channel of the co-staining antibody were obtained, and background signals for each channel were subtracted from the measurements. For each condition, 10 kinetochores in at least 10 prometaphase cells were measured. Fluorescence intensity measurements were normalized to the CREST signals, and the average and standard deviation (s.d.) were plotted.

### Metabolic Cell Labeling

HeLa cells were synchronized using 100 ng/ml nocodazole for 16 h. For the last 4 h, cells were incubated with phosphate free DMEM supplemented with 5% FBS (dialyzed to remove phosphates) in the continued presence of nocodazole. Cells were then labeled with 330 μCi ^32^P orthophosphate (5 mCi/ml H3PO4, MP Biomedicals) in 1 ml of phosphate free DMEM plus 5% FBS for another 4 h in the presence of nocodazole. Half of the mitotic cells were also incubated with 2 μM ZM447439 to inhibit Aurora B kinase activity. Cells were harvested and immunoprecipitated with 1 μl rabbit anti-NudC 70/1 antibody [32]. Immunoprecipitated proteins were resolved on a 4–12% gradient gel, transferred to a nitrocellulose membrane, subjected to autoradiography and then immunoblotted with goat anti-NudC peptide antibody [33].

### Statistical Analysis

A student’s t-test was performed on sample sets with two groups and ANOVA was performed on sample sets with more than three independent groups. p values less than 0.05 were considered to be statistically significant.

## Results

### NudC Colocalizes with Aurora B at the Midbody and Co-Immunoprecipitates with Aurora B in Mitosis

**Fig 1:**
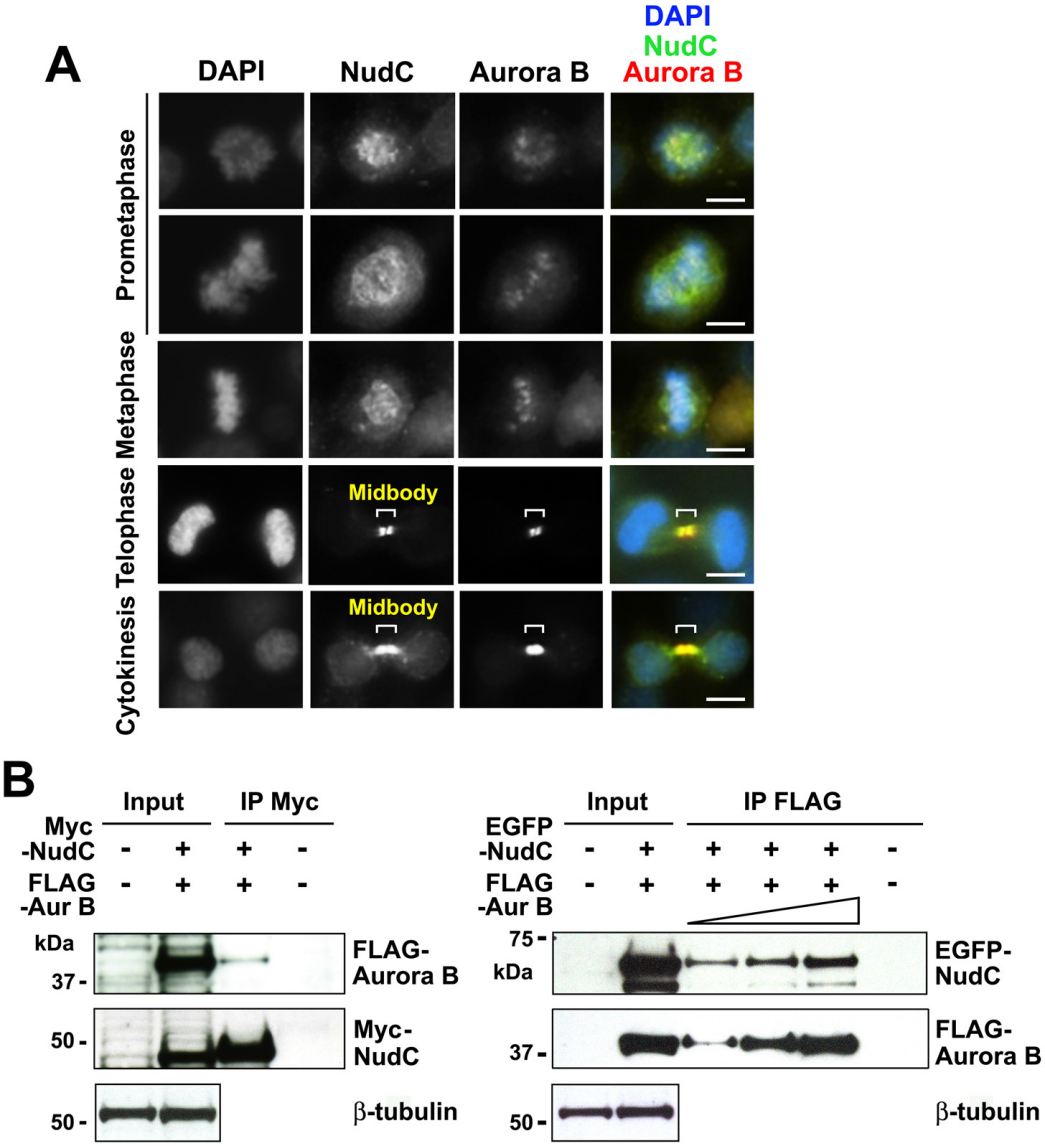
NudC co-localizes with Aurora B in mitosis. (A) Unperturbed mitotic HeLa cells were stained for NudC (green), Aurora B (red) and counterstained with DAPI (blue). Bar, 10 μm. (B) HeLa cells were transfected with Myc-NudC and FLAG-Aurora B (left) or EGFP-NudC and FLAG-Aurora B (right) for 24 h. Cell lysates (1 mg in 250 μl) were immunoprecipitated with anti-Myc antibody and blotted for Aurora B followed by reblotting for NudC (left). A reciprocal immunoprecipitation was performed, in which cell lysates (500 μg in 250 μl, 1 mg in 250 μl or 2 mg in 500 μl) were immunoprecipitated with anti-FLAG antibody followed by blotting for NudC and reblotting for Aurora B (right). Immunoprecipitation with either anti-Myc or anti-FLAG antibody using non-transfected cell lysates was used as a negative control. β-tubulin was used as a loading control. Input, 20 μg total cell lysates. Data are representative of n = 5 independent experiments.

To determine if NudC associates with Aurora B during mitosis, we examined whether the two proteins colocalize at mitotic structures. Using indirect immunofluorescence, we found that NudC and Aurora B co-localize at the kinetochore in prometaphase and metaphase cells in early mitosis (Fig 1A). As cells progress into late mitosis, NudC and Aurora B co-localize as two foci at the midbody located in between the two dividing daughter cells (Fig 1A). The central region of the midbody is not accessible to antibodies due to the an electron-dense matrix of unknown composition [42,43] and thus appears as a “dark zone” [2]. To examine if NudC interacts biochemically with Aurora B, we first co-transfected cells with Myc-NudC and FLAG-Aurora B [44] for 24 h. By immunoprecipitation of NudC with anti-Myc antibody, we found that FLAG-Aurora B was present in the NudC immunoprecipitated complex (Fig 1B, left). We next co-transfected EGFP-NudC and FLAG-Aurora B and performed a reciprocal immunoprecipitation and found that immunoprecipitation of Aurora B with anti-FLAG antibody brought down EGFP-NudC (Fig 1B, right). As negative controls, we performed immunoprecipitations using anti-tag antibodies with lysates from non-transfected cells (Fig 1B). The absence of Aurora B from the anti-Myc immunoprecipitation or NudC from the anti-FLAG immunoprecipitation showed the specificity of NudC association with Aurora B and vice versa. Taken together, our studies show that NudC interacts with Aurora B in an immunoprecipitable complex, in addition to their co-localization on mitotic structures in mitotic cells.

### NudC Shows Binding to Aurora B in Mitosis

**Fig 2:**
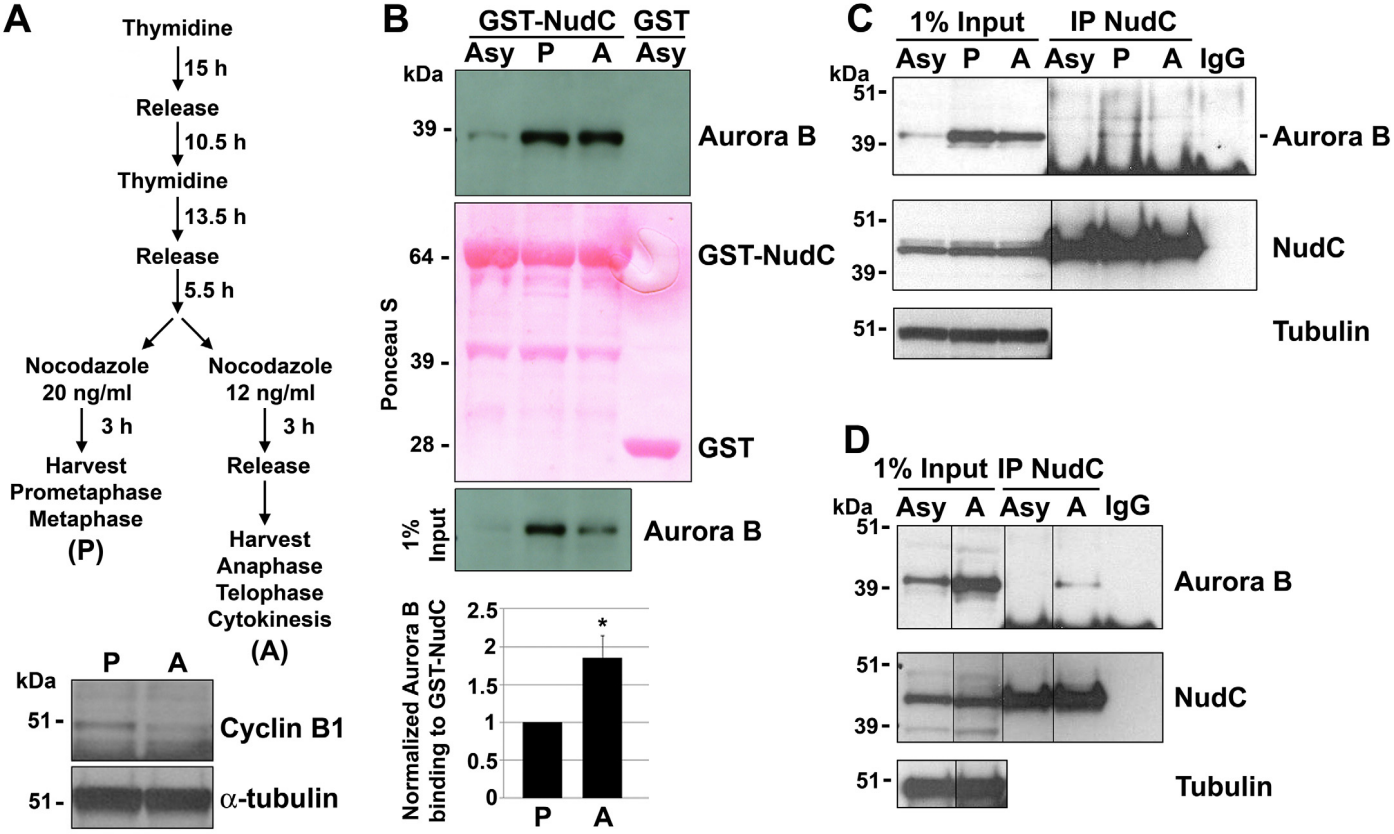
NudC interaction with Aurora B in mitosis. (A) HeLa cells were synchronized by a double thymidine block and release protocol as indicated. “P” (prometaphase and metaphase) and “A” (anaphase, telophase and cytokinesis) lysates were prepared from early versus late mitotic cells. Synchronization efficiency was confirmed by a cyclin B1 western blot. α-tubulin was used as a loading control. (B) Lysates from asynchronously cycling (Asy), P or A cells were incubated with GST-NudC fusion protein in GST pulldown assays. GST-NudC bound proteins were immunoblotted for Aurora B. GST binding to lysates from either Asy (this experiment), P or A cells (not shown), served as a negative control. Ponceau S staining showed equal GST-NudC fusion protein used in the pulldown assay. Aurora B binding was quantified as Aurora B signal/input Aurora B normalized against the P sample (mean ± s.e.m.) from 3 independent experiments. *, p < 0.05. (C) Lysates (2 mg in 500 μl) from Asy, P or A cells prepared as in (A) were immunoprecipitated with G1 goat NudC antibody, blotted for Aurora B and reblotted for NudC using 2D9 monoclonal antibody. Asy lysates were also immunoprecipitated with preimmune goat serum (IgG) as a negative control. β-tubulin was used as a loading control. (D) An immunoprecipitation using a different batch of A cell lysate (500 μg in 250 μl) was performed as in (C).

To analyze at which stage in mitosis NudC interacts with Aurora B, we synchronized HeLa cells using a double thymidine block and release protocol followed by nocodazole treatment to enrich for cells in early mitosis, including prometaphase and metaphase (labeled as “P”), versus late mitosis, including anaphase, telophase and cytokinesis (labeled as “A”) [45] (Fig 2A). Immunoblot of cyclin B1 confirmed that the cells were properly synchronized, as cyclin B1 levels peak in metaphase and decline during metaphase-to-anaphase transition due to degradation by the APC/C proteasome pathway (Fig 2A) [46]. Similarly, total Aurora B protein levels were found to decline as expected at the metaphase-to-anaphase transition (Fig 2B, input). We found that Aurora B binds GST-NudC in both early and late mitosis in GST-NudC pulldown assays (Fig 2B). We further examined endogenous NudC interaction with Aurora B in early and late mitosis, using P and A cell lysates prepared as in (Fig 2A), in co-immunoprecipitation assays. We detected Aurora B in the NudC immunoprecipitated complex in both the P and A lysates, but not in the Asy lysates (Fig 2C). Although the Aurora B signal was weak, it was reproducibly observed in the NudC immunoprecipitated complex using a different batch of A cell lysates (Fig 2D). The absence of Aurora B when preimmune serum (IgG) was used in the immunoprecipitation showed the specificity of Aurora B interaction with NudC (Fig 2C and 2D). These studies show that NudC and Aurora B associate in both early and late stages of mitosis.

### NudC is a Substrate of Aurora B

**Fig 3:**
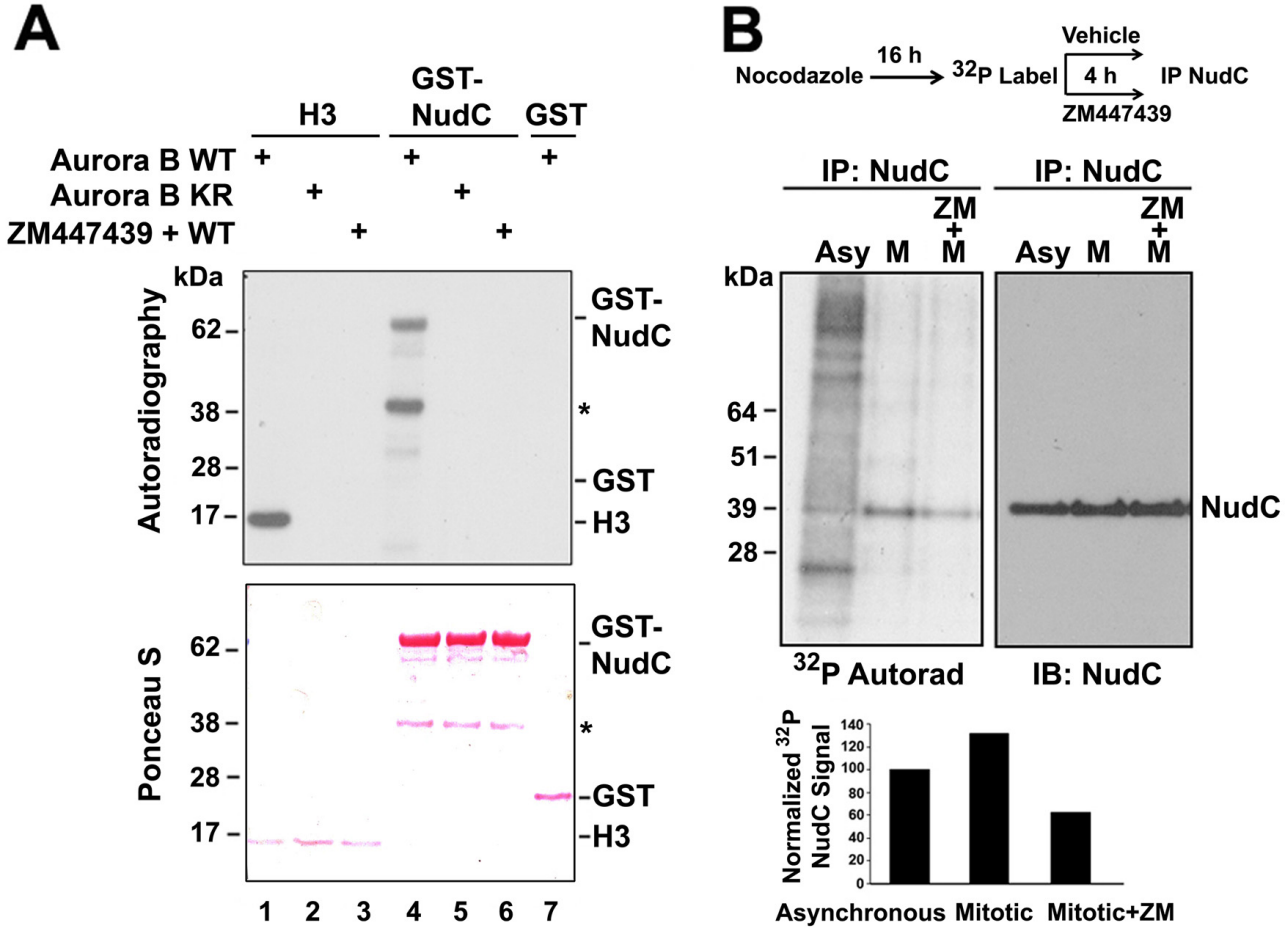
NudC is phosphorylated by Aurora B *in vitro* and *in vivo*. (A) HeLa cells were transfected with FLAG-Aurora B wild type (WT) or a kinase dead (K106R) mutant Aurora B for 24 h. Aurora B was immunoprecipitated using anti-FLAG antibody and used in IP kinase assays. Substrates used were GST-NudC (lanes 4–6), histone H3 (lanes 1–3) as a positive control, and GST (lane 7) as a negative control. Aurora B WT was also incubated with 2 μM of ZM447439 as a specificity control (lanes 3 and 6). Samples were transferred to a filter, stained by Ponceau S (lower panel) and analyzed by autoradiography (upper panel). *, degradation product. Data are reproducible in 3 independent experiments. (B) HeLa cells were synchronized by an overnight incubation with 100 ng/ml nocodazole (M, mitotic) as indicated. Cells (1 X 10^6^) were labeled with ^32^P orthophosphate for 4 h in the presence or absence of 2 μM ZM447439 (ZM). Cell lysates (300 μg at 1 mg/ml) were immunoprecipitated for NudC, transferred to a filter, analyzed by autoradiography, and immunoblotted for NudC. ^32^P-NudC was quantified as ^32^P-NudC/total immunoprecipitated NudC and normalized against NudC signals in asynchronously cycling (Asy) cells.

Given the interaction between NudC and Aurora B, we asked if NudC might be phosphorylated by Aurora B. HeLa cells were transfected with either wild-type FLAG-Aurora B (WT) or a kinase-dead (K109R) mutant FLAG-Aurora B [44], along with the Aurora B coactivator/CPC scaffold protein Myc-INCENP [47], for 24 h. Aurora B was then immunoprecipitated with anti-FLAG antibody. Incubation of GST-NudC with wild type (WT) or kinase-dead (KR) Aurora B showed that GST-NudC was phosphorylated by WT Aurora B (Fig 3A, lane 4) but not by kinase-dead mutant Aurora B (Fig 3A, lane 5). The lower molecular weight band observed in the GST-NudC lanes (Fig 3A, lanes 4–6, asterisk) is likely a breakdown product. The phosphorylation of NudC by Aurora B was inhibited by the small molecule inhibitor ZM447439 [48–50] (Fig 3A, lane 6), indicating that the phosphorylation of NudC is due to Aurora B kinase activity. An IP kinase assay performed with Aurora A did not generate phosphorylated NudC (data not shown), further supporting that the phosphorylation of NudC by Aurora B is specific. Histone H3, a well-known substrate of Aurora B, was used as a positive control (Fig 3A, lane 1), while GST alone served as a negative control (Fig 3A, lane 7). Note that under our experimental condition, Aurora B autophosphorylation signal is weaker than signals from Aurora B substrate phosphorylation of H3 and GST-NudC, and thus it is not visible in the IP kinase assays. These results show that NudC is a substrate of Aurora B *in vitro*.

We further examined whether NudC is phosphorylated by Aurora B *in vivo*. Asychronoulsy cycling (Asy) HeLa cells as well as cells synchronized in mitosis (M) were pulse-labeled with ^32^P-orthophosphate for 4 h. ^32^P-labeled NudC was immunoprecipitated with an anti-NudC antibody, analyzed by autoradiography following transfer to a filter, and further immunoblotted for total NudC. We found that the phosphorylation level of endogenous NudC was higher in mitotic cells compared with that in asynchronously cycling cells (Fig 3B). When mitotic cells were treated with the Aurora B inhibitor ZM447439, there was ~ 60% decrease in ^32^P-labeled NudC compared to that in the mitotic population, suggesting that NudC is phosphorylated by Aurora B during mitosis. Together, these results show that NudC is an *in vitro* and *in vivo* substrate of Aurora B.

### NudC Is Phosphorylated by Aurora B on Residue T40

**Fig 4:**
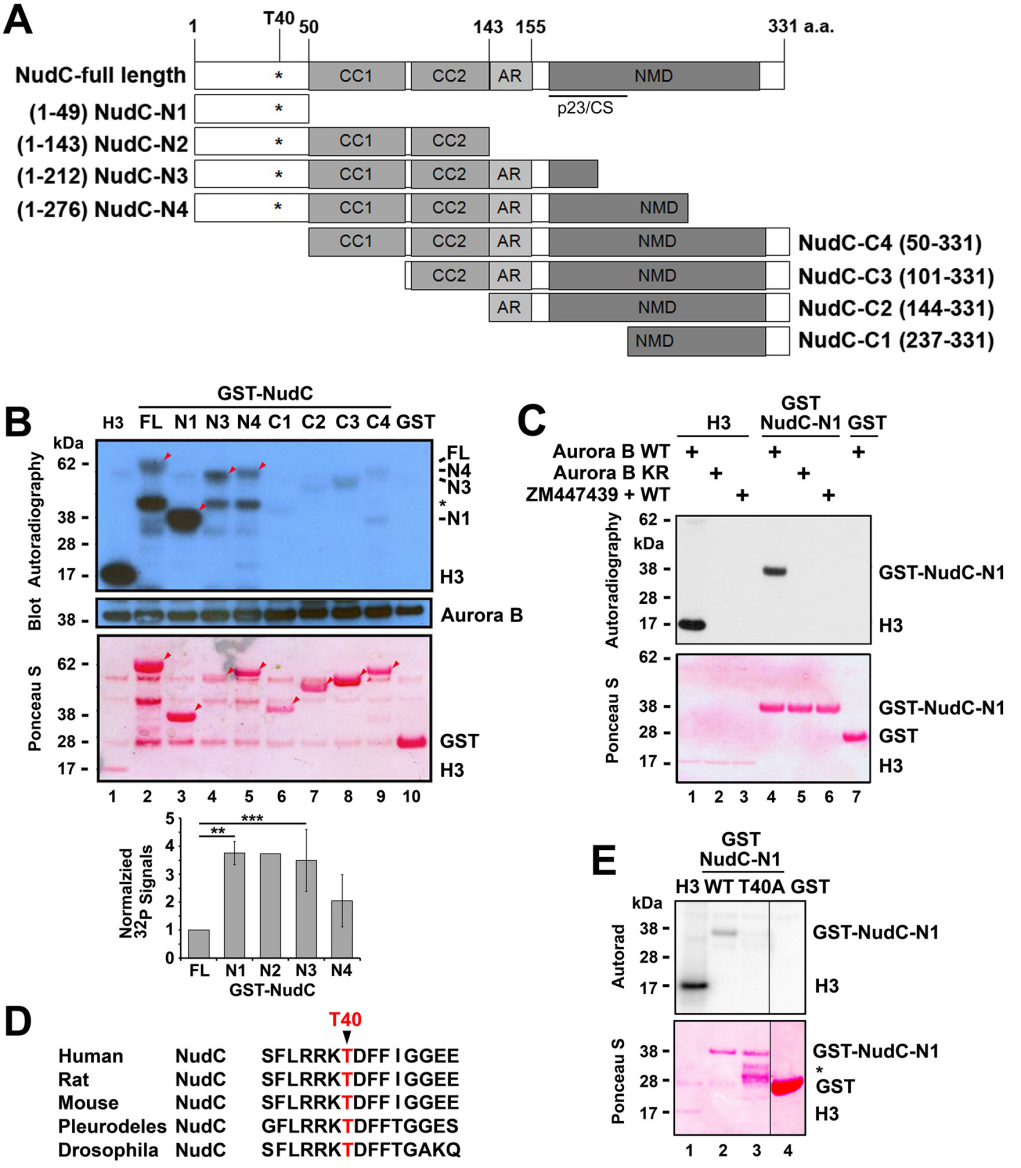
NudC is phosphorylated by Aurora B on T40. (A) A series of GST-NudC truncations were constructed based on functional domains in human NudC. N1 –N4, NudC truncations that retain the N terminal 49 amino acids (a.a.) but contain various deletions from the C terminus. C1 –C4, NudC truncations that retain most or the entire C terminal nuclear movement domain but contain various deletions from the N terminus. Numbers within brackets refer to amino acid residues in the human NudC protein. CC, coiled-coiled; AR, acidic rich; p23-like CHORD-Sgt domain [51]; NMD, conserved nuclear movement domain. (B) GST-NudC full-length (FL), N- and C- terminal truncation series depicted in (A) were used in Aurora B IP kinase assays. Reactions were transferred to filters, analyzed by autoradiography, blotted for Aurora B, and stained by Ponceau S. Substrates used were GST-NudC (lanes 2–9), histone H3 (lane 1) as a positive control and GST (lane 10) as a negative control. Arrowheads, ^32^P-labeled GST-NudC proteins in the autoradiogram corresponding to the GST-NudC proteins in the Ponceau stain. *, degradation product. The levels of ^32^P-GST-NudC signals (autoradiogram)/total GST-NudC (Ponceau) normalized against that of GST-NudC full-length (set as 1) were quantified (mean ± s.e.m.) from 3 independent experiments, except for GST-NudC-N2 which was obtained from one experiment (data not shown). **, p < 0.001; ***, p < 0.04. (C) GST-NudC-N1 was used in Aurora B IP kinase assays. (D) NudC protein sequences from various species share a high degree of sequence homology surrounding amino acid T40. (E) GST-NudC-N1 wild type (WT) and GST-NudC-N1 containing T40A mutation were used in Aurora B IP kinase assays. GST, negative control. Data in C and E are representative of 3 independent experiments.

We determined Aurora B phosphorylation site(s) on NudC using a series of GST-NudC truncations. The truncations were generated to delete potential functional domains in NudC, including coiled-coil domains 1 and 2 (CC1 and CC2), an acidic residue-rich domain (AR), the p23-like CHORD-Sgt (p23/CS) domain [51] and the nuclear movement domain [14,18,32,52], from either the C terminus (NudC-N1 to NudC-N4) or the N terminus (NudC-C1 to NudC-C4) (Fig 4A). We found that all of the NudC truncations that contained the N terminal 49 amino acids were phosphorylated by Aurora B in IP kinase assays (Fig 4B, lanes 2–5, arrowheads). GST-NudC-N1, containing the first 49 amino acid of NudC, was efficiently phosphorylated by Aurora B (Fig 4B, lane 3). Quantification showed that GST-NudC-N1 and GST-NudC-N2 exhibited a ~4-fold increase in ^32^P labeling relative to that in the GST-NudC full-length protein (Fig 4B, lower panel). GST-NudC-N3 and GST-NudC-N4 truncations showed a decline in phosphorylation by Aurora B, suggesting that protein sequences within the nuclear movement domain at the C terminus of NudC may block Aurora B phosphorylation of the N terminus of NudC. All of the NudC truncations that lacked the N terminal 49 amino acids were poorly phosphorylated by Aurora B (Fig 4B, lanes 6–9). Taken together, these data suggest that the N terminus of NudC may contain a major Aurora B phosphorylation site. Thus, we focused on analyzing the N terminus of NudC.

The specificity of Aurora B phosphorylation of GST-NudC-N1 was examined by using kinase-dead Aurora B or ZM447439 in an IP kinase assay. GST-NudC-N1 was found to be phosphorylated by wild-type Aurora B (Fig 4C, lane 4), but not by kinase-dead Aurora B (Fig 4C, lane 5) or wild-type Aurora B that was treated with the inhibitor ZM447439 (Fig 4C, lane 6). Similar to Fig 3, histone H3 served as a positive control (Fig 4C, lane 1) while GST served as a negative control (Fig 4C, lane 7). These results indicate that the N terminus of NudC contains an Aurora B phosphorylation site.

Sequence analysis of the N terminus of NudC revealed a sequence FLRRKTDFF that is evolutionarily conserved from Drosophila to man [17] (Fig 4D), where the RKT motif has been shown to be phosphorylated by Aurora B in other proteins [12,53,54]. Site-directed mutagenesis was performed to mutate the T40 residue to generate T40A phospho-defective or T40D phospho-mimetic NudC mutants. Using the GST-NudC-N1 T40A mutant in an IP kinase assay, we found that GST-NudC-N1 wild-type was phosphorylated by Aurora B (Fig 4E, lane 2) but not the GST-NudC-N1 T40A mutant (Fig 4E, lane 3). Thus, T40 in NudC is an Aurora B phosphorylation site.

### NudC Knockdown Does Not Affect Aurora B Localization at the Kinetochore

**Fig 5:**
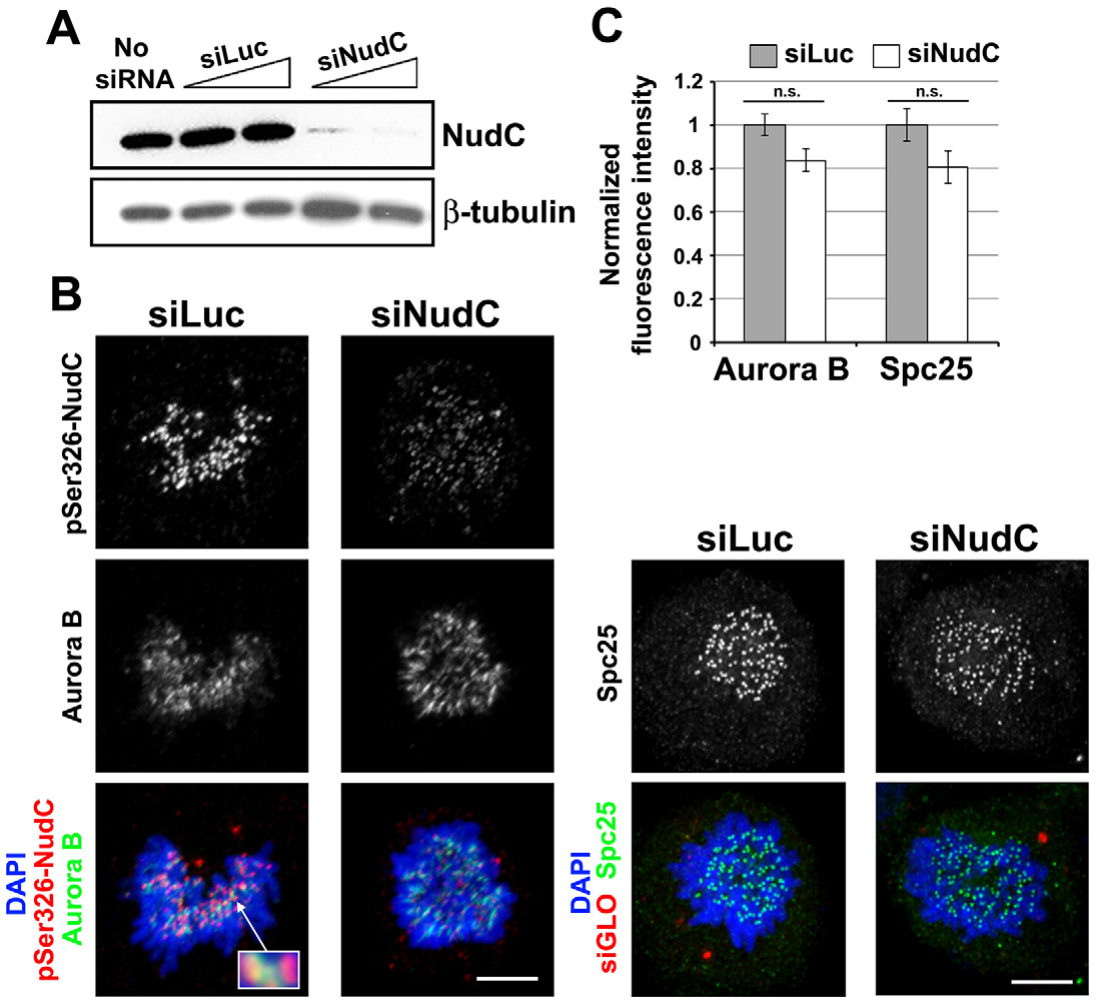
Aurora B localization at the kinetochore is not affected in NudC-deficient cells. (A) HeLa cells were transfected with siLuc or siNudC oligos for 72 h. NudC knockdown was examined by western blotting for NudC. β-tubulin was used as a loading control. (B) Prometaphase cells treated with siRNAs as in (A) were stained for pS326-NudC (red) and Aurora B (green) (enlarged in inset), or with Spc25 (green), and counterstained with DAPI for DNA (blue). In initial experiments, siGLO was co-transfected as an indicator for siRNA oligo uptake. (C) For quantification, cells treated as in (B) were also co-stained with the CREST autoserum to mark the kinetochores. For Aurora B or Spc25 staining, maximum-intensity projections of deconvolved images were measured using AutoDeblur/AutoVisualize software, and their fluorescence intensities (average ± s.d.) relative to that of CREST staining at the kinetochore were quantified, using 10 randomly chosen kinetochores from at least 10 siLuc or siNudC prometaphase cells. n.s., not significant.

Given that NudC interacts with Aurora B (Figs 1 and 2), we asked whether NudC plays a role in Aurora B localization in early mitotic cells, where Aurora B is localized on kinetochores during prometaphase [7,9,39,55]. To examine this, HeLa cells were transfected with Luciferase or NudC siRNA oligos for 72 h. Western blot analysis showed a reduction of NudC protein levels in siNudC cells but not in siLuc control cells (Fig 5A). Next, siLuc and siNudC cells undergoing unperturbed mitosis were stained for NudC and Aurora B. Using pSer326-NudC antibody that specifically recognizes NudC at the kinetochore [32], we found that NudC staining at the outer kinetochore flanked that of Aurora B staining at the inner kinetochore (Fig 5B, inset). NudC knockdown resulted in a depletion of NudC signals at the kinetochore while Aurora B staining at kinetochores remained unchanged in siNudC cells relative to that in siLuc cells (Fig 5B). As a control, we examined the presence of another outer kinetochore protein Spc25, a subunit of the kinetochore-based Ndc80 complex [37], at the kinetochore. To quantify the levels of Aurora B and Spc25 at the kinetochore, cells were co-stained with the human CREST autoserum to identify the inner region of kinetochores. We then measured the relative fluorescence intensity of Aurora B or Spc25 normalized to that of CREST staining at individual kinetochores using confocal microscopy. The levels of Aurora B and Spc25 at the kinetochores were not significantly affected by NudC depletion in prometaphase cells (Fig 5C). These results show that NudC knockdown does not affect Aurora B localization at the kinetochore in early mitosis. This finding led us to focus on NudC interaction with Aurora B at later stages of mitosis.

### NudC Knockdown Leads to Elongated Intercellular Bridge and Sustained Aurora B Activity in the Midbody

**Fig 6:**
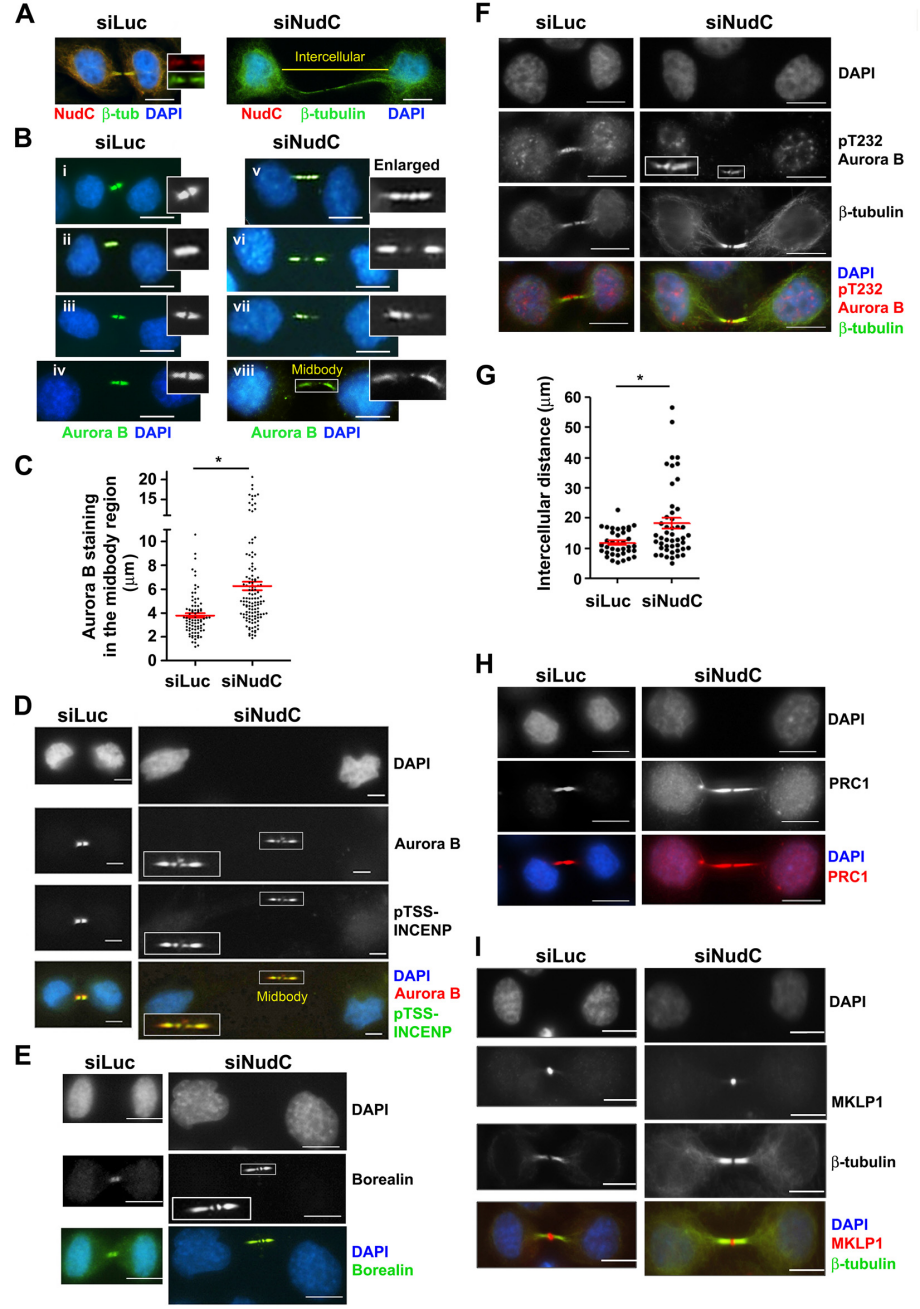
Aurora B distribution in the midbody region in NudC-deficient cells. (A) HeLa cells were transfected with either siLuc control or siNudC oligos for 72 h. Pairs of interconnected cells undergoing unperturbed cytokinesis were stained for NudC (red), tubulin (green), and counterstained with DAPI (blue). Midbody is enlarged in control cells. Staining with two NudC antibodies, G1 goat or 2D9, gave similar results. Yellow line depicts intercellular distance. (B) Pairs of interconnected siLuc (i–iv) or siNudC (v–viii) cells undergoing cytokinesis were stained for Aurora B (green) and counterstained with DAPI (blue). Midbodies are enlarged in insets. (C) Aurora B staining in the midbody region in siLuc control and siNudC cells in (B) was measured using Nikon NIS-Elements software, and the lengths in μm (mean ± s.e.m.) are presented using GraphPad Prism software. The numbers of midbodies counted in siLuc (n = 84) and siNudC (n = 121) cells were obtained from 3–5 independent experiments. *, p < 0.01. siLuc and siNudC cells were also stained as follows: (D) Aurora B (red) and pTSS-INCENP (green). (E) Borealin (green). (F) pT232 Aurora B (red) and β-tubulin (green). Midbodies are enlarged in insets. (H) PRC1 (red). (I) MKLP1 (red) and β-tubulin (green). All bars, 10 μm. (G) Intercellular distances between interconnected daughter cells were determined by staining with tubulin, as indicated by the yellow line in (A). The lengths in μm (mean ± s.e.m.) were obtained from siLuc (n = 37) and siNudC (n = 49) cells in n = 2 experiments. *, p < 0.01.

It is known that Aurora B localizes to the midzone and midbody to regulate cleavage furrow formation [56,57] and cell abscission [6], respectively. We examined Aurora B localization at the cleavage furrow and midzone in anaphase cells, but did not observe defects in Aurora B localization at these sites following NudC knockdown (data not shown). We previously showed that NudC knockdown leads to an increase in cells connected with intercellular cytoplasmic bridges and multinucleation, suggesting a failure in cytokinesis [34,35]. We next examined the interaction of NudC and Aurora B at the midbody in the final stages of mitosis. HeLa cells were transfected with either Luciferase or NudC siRNA oligos for 72 h, and cells in unperturbed cytokinesis were examined by staining for NudC. While control cells showed NudC staining in the intercellular bridge (Fig 6A, left, inset), knockdown of NudC resulted in a loss of NudC signals in the elongated intercellular bridge (Fig 6A, right). In siLuc control cells, Aurora B is seen as either “two dots” separated by the dark zone (Fig 6Bi and 6Bii, insets) in earlier-stage cytokinetic cells with more condensed DNA or “two whiskers” (Fig 6Biii and 6Biv, insets) in later-stage cytokinetic cells with less condensed DNA. In contrast, an unusual pattern of Aurora distribution was observed at the midbody in NudC-deficient cells. In addition to being found as two foci at the midbody, Aurora B staining was also found to be distributed past the lateral constriction zone (narrowed region past the midbody) into the flanking regions beyond [42,43] in NudC-deficient cells (Fig 6Bv–6Bviii, insets). Note that the unusual Aurora B staining pattern could be observed in NudC-deficient cells that had retained the normal distance between divided cells (Fig 6Bv, inset) as compared with that in siLuc cells (Fig 6Bi and 6Bii, insets). Aurora B staining could also be observed to be asymmetrically distributed in the flanking region to one side of the midbody in NudC-deficient cells (Fig 6Bvii and 6Bviii, insets). We next measured the length of the midbody region positive for Aurora B staining. In siLuc control cells, Aurora B staining at the midbody was found to be around 4 μm in length (Fig 6C), a median midbody length observed in several cell types [2,42,58]. In contrast, in NudC-deficient cells Aurora B staining was found to spread out to a median length of 6.5 μm, with lengths reaching up to 15–20 μm in some cells (Fig 6C).

We next examined the localization of other components of the CPC, including INCENP and Borealin [7,9] at the midbody. In siLuc control cells, pTSS-INCENP (Fig 6D, left) and Borealin (Fig 6E, left) are localized as two foci separated by the dark zone at the midbody. Following NudC knockdown, INCENP and Borealin were also found to be distributed past the lateral constriction zone into the flanking regions beyond [42,43] (Fig 6D and 6E, right, insets). These observations suggest that the Aurora B/CPC complex is aberrantly localized that the midbody region in NudC-deficient cells.

Despite its unusual distribution beyond the midbody, Aurora B remained functionally active in the elongated intercellular bridge in NudC-deficient cells, as evidenced by the presence of Aurora B substrate phosphorylation on pTSS-INCENP^834-902^ that promotes maximal Aurora B activity [36,59] (Fig 6D, right, inset) and Aurora B autophosphorylation on pT232 [60] (Fig 6F, right, inset). It has been suggested that the presence of chromatin in the midzone/midbody may trigger Aurora B activation, as part of the abscission checkpoint to delay abscission until the chromatin bridge can be resolved [6,61,62]. However, we did not detect chromatin in the elongated midbody region in the majority of NudC-deficient cells (Fig 6). Strikingly, NudC depletion resulted in a significant increase in the length of the intercellular bridge connecting daughter cells, from 11.9 (± 0.7) μm in control cells, a length commonly observed in HeLa cells [63], to 18.4 (±1.7) μm or longer distances reaching up to 56 μm in NudC-deficient cells (Fig 6A and 6G). Taken together, NudC depletion resulted in elongated intercellular bridge, unusual spreading of Aurora B beyond the midbody into the intercellular region, and sustained Aurora B activity in the elongated intercellular bridge.

### NudC Knockdown Does Not Affect the Localization of Microtubule Organizing Proteins PRC1 and MKLP1

The midbody is composed of tightly packed, interdigitating anti-parallel midzone microtubules, microtubule bundling protein PRC1 [64,65], kinesin motors MKLP1 [1,2,56,66,67], MKLP2 [67–69] and KIF4 [64,70,71], and endosomal trafficking proteins that provide membranes for cell abscission [43,62,72,73]. We next examined PRC1 and MKLP1, which are two proteins that do not require Aurora B for their localization to the midbody and which exhibit distinct localization patterns as compared to the CPC at the midbody. The microtubule bundling protein PRC1 was found to be distributed along the length of the intercellular bridge, as expected, in both the control and NudC-deficient cells (Fig 6H). The kinesin motor MKLP1, usually found as a ring structure at the midbody [2,56], was found as a single focus at the midbody in both control and NudC-deficient cells (Fig 6I). These results suggest that NudC does not play a role in the localization of PRC1 or MKLP1 at the midbody.

### Wild-Type NudC but Not T40 Phosphorylation Mutant NudC Rescues Cytokinesis

**Fig 7:**
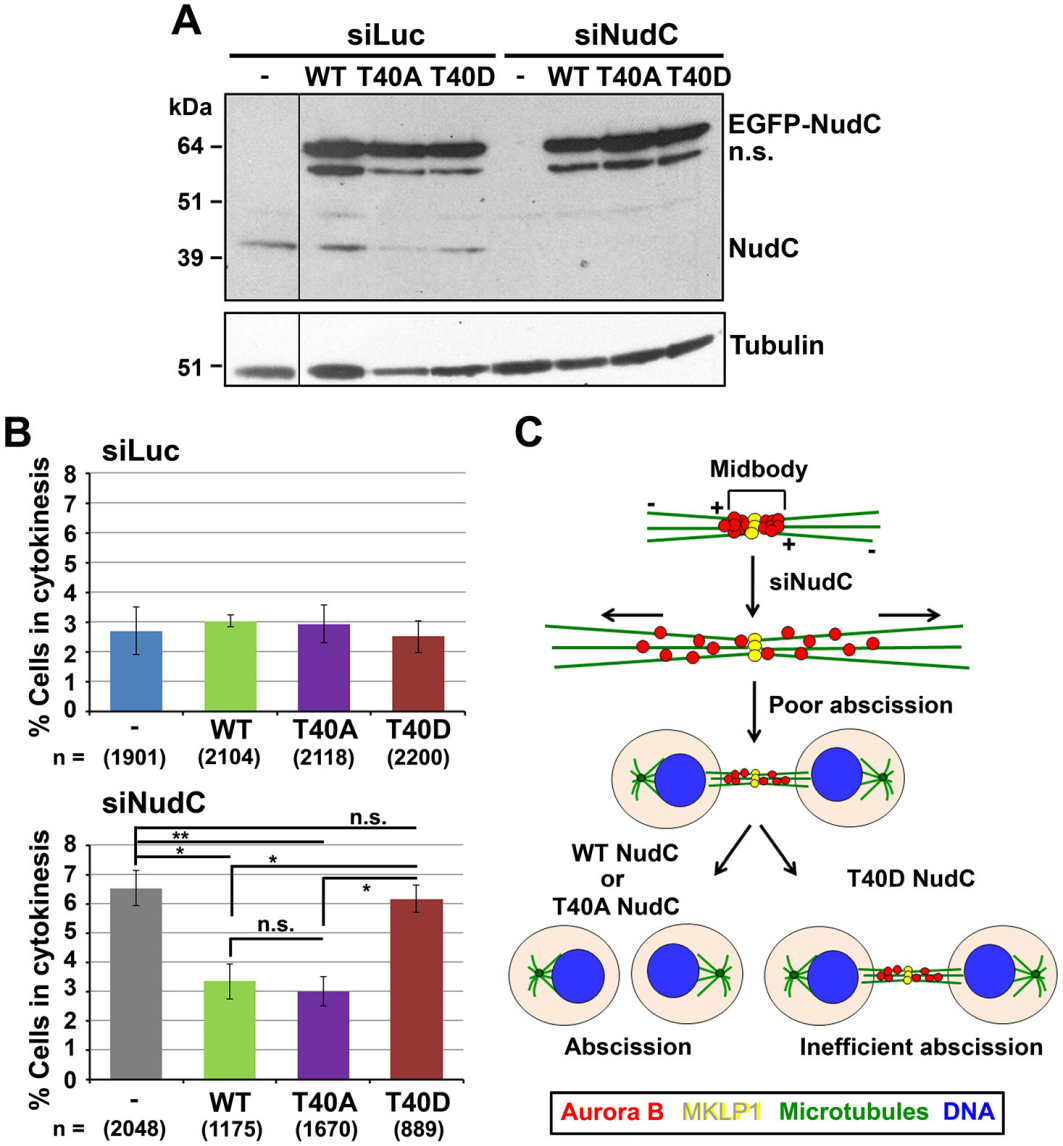
Dynamic phosphorylation of NudC on T40 by Aurora B regulates cytokinesis. (A) HeLa cells were transfected with siLuc or siNudC oligos for 48 h. Cells were further transfected with EGFP-NudC WT, T40A or T40D for another 24 h. Cell lysates (5 μg) were blotted for NudC followed by β-tubulin as a loading control. n.s., nonspecific band. (B) Cells prepared as in (A) that demonstrated a connection by an intercellular bridge were analyzed. % cells in cytokinesis (mean ± s.e.m.) was determined from 3–5 independent experiments. n, number of cells counted. Statistical significance was calculated using ANOVA. *, p < 0.05; **, p < 0.01. (C) Model of NudC phosphorylation on T40 by Aurora B on cytokinesis and cell abscission. In NudC knockdown cells, Aurora B is widely distributed at the midbody region. This is correlated with over-extension of microtubules (horizontal arrows) in the elongated intercellular bridge and poor cell abscission. Reconstitution with wild-type (WT) NudC or unphosphorylatable T40A NudC rescues abscission and cytokinesis. Reconstitution with the phospho-mimetic T40D NudC is inefficient in completing abscission and cytokinesis. The model suggests that dynamic phospho-regulation on NudC T40 by Aurora B is important in regulating cell abscission and cytokinesis.–, microtubule minus-ends; +, microtubule plus-ends. Horizontal arrows, microtubule sliding and elongation in the intercellular bridge.

Cytokinesis completion requires the cleavage of the midbody to separate the daughter cells [5,6,10]. We examined whether Aurora B phosphorylation of NudC at T40 plays a role in cytokinesis. To address this, we performed NudC knockdown followed by reconstitution with the T40 phosphorylation NudC mutants. Western blot analysis showed knockdown of endogenous NudC in siNudC-treated cells and equal expression of exogenous EGFP-NudC wild-type (WT), T40A or T40D mutants in siLuc control as well as siNudC cells after transfection (Fig 7A). Next, GFP-positive cells undergoing cytokinesis were analyzed. Expression of GFP-NudC WT or T40 mutant constructs did not have a significant effect on cytokinesis in control siLuc cells (Fig 7B, upper). In contrast, we observed a significant increase (p < 0.01) in cells connected by an intercellular bridge in NudC-deficient cells relative to that in siLuc control cells, suggesting problems in cytokinesis (Fig 7B, lower). Exogenous expression of WT NudC was able to significantly rescue cytokinesis in siNudC cells to a level observed in siLuc control cells. Interestingly, the phosphorylation-defective T40A NudC mutant was also able to rescue cytokinesis. In contrast, the phosphorylation-mimetic T40D NudC mutant was found to be inefficient in completing cytokinesis (Fig 7B, lower). Taken together, these results suggest that both NudC levels and its dynamic phosphorylation on T40 by Aurora B play a role in cell abscission that occurs at the end of cytokinesis.

## Discussion

Our studies show that NudC is an Aurora B substrate, and suggest that NudC phosphorylation by Aurora B plays a role in regulating cell abscission during cytokinesis.

### NudC Regulates Aurora B Distribution at the Midbody

The Aurora B/CPC complex is targeted to specific locations at different stages of mitosis and cytokinesis, and mediates mitotic progression through phosphorylation of various substrates [11]. NudC is one of many proteins identified in the midbody proteome [74] and plays a role in regulating cytokinesis [34,35]. We found that NudC interacts with and co-localizes with Aurora B at the midbody in late mitosis, and is phosphorylated by Aurora B on pT40. NudC appears to restrict Aurora B distribution to the midbody, as elongated midbodies with Aurora B/CPC positivity beyond the midbody region was observed in NudC-deficient cells. Several possibilities may explain this phenotype. NudC may be involved in localizing the Aurora B/CPC complex to the midbody, as NudC is an associated factor of the dynein-dynactin motor complex [14,18,20,22,75], and has been shown to mediate microtubule plus-end-directed cargo transport in neuronal cells [22,23,27], nuclear movement along the hyphae in the filamentous fungus *Aspergillus nidulans* [14,18,20,25], and apical nuclear migration in radial glial progenitor cells during neocortical brain development [26]. The lack of NudC may thus hamper Aurora B/CPC concentration to the microtubule plus-ends at the midbody. Alternatively, NudC contains a p23-like CHORD-Sgt domain similar to small heat shock proteins [76] and has been shown to exhibit chaperone activity *in vitro* [51,52,77,78]. In this context, insufficient levels of NudC may lead to the spreading of Aurora B/CPC in the elongated midbody in NudC-deficient cells.

### NudC Regulates Intercellular Bridge Elongation

The presence of extraordinarily long intercellular bridges is a striking feature in NudC-deficient cells [34,35] (Fig 6). Newly-identified functions of Aurora B/CPC in budding yeast may in part explain this phenotype. Aurora B/CPC localization along anaphase spindles has been suggested to promote microtubule plus-end polymerization, while their concentration at the midbody is shown to slow down microtubule polymerization to prevent spindle overgrowth [79]. The CPC also controls spindle elongation through the kinesin-5 motor [80], where kinesin-5 can switch between a force generator that promotes outward sliding of spindle microtubules or a brake to inhibit spindle elongation depending on CPC activity. It has been shown that Aurora B can reach its targets through a diffusion-based kinase activity gradient, as has been observed for Aurora B at the centromere [81–83], spindle midzone [84], and spindle microtubules [85]. The aberrant spreading of active Aurora B (pT232)/CPC (pTSS-INCENP) beyond the midbody may generate an even more extended activity gradient to reach targets further along the intercellular bridge. One such target might be the microtubule depolymerizing kinesin KIF2A that regulates microtubule lengths at their minus ends [63]. It is possible that continued phosphorylation of KIF2A by an extended Aurora B kinase gradient may inhibit the depolymerizing activity of KIF2A at microtubule minus-ends, thus contributing to the over-extension of microtubules in the elongated intercellular bridge in NudC-deficient cells.

### NudC in Cytokinesis Regulation

At the end of cytokinesis, a decline in Aurora B activity together with dephosphorylation of Aurora B substrates is required for the destabilization of the intercellular bridge and completion of cell abscission [5,6,8,43,62]. We envisage that NudC is phosphorylated at T40 by Aurora B when the Aurora B-mediated abscission checkpoint is turned on, and that NudC is dephosphorylated when the abscission checkpoint is turned off. Such a scenario could explain our findings that overexpression of the phospho-mimetic T40D NudC (abscission checkpoint on) is correlated with inefficient abscission and cytokinesis failure, while the unphosphorylatable T40A NudC (abscission checkpoint off) is able to rescue cytokinesis in NudC-deficient cells (Fig 7B). We suggest that a balance between phosphorylated and dephosphorylated T40 NudC plays a role in regulating cell abscission at the end of cytokinesis (Fig 7C).

### Post-Translational Modification of NudC in Mitosis and Cytokinesis

The mitotic functions of NudC is regulated by post-translational modifications, including deacetylation by HDAC3 [33] and phosphorylation by at least three mitotic kinases, including Cdk1 (data not shown), Plk1 [32,35] and Aurora B (this study). While the function of Cdk1-phosphorylated NudC is not known, Plk1 phosphorylated S274/S326 NudC is involved in recruiting Plk1 to kinetochores to regulate kinetochore-microtubule attachments in early mitosis [32] and localizing Plk1 to the midzone/midbody to regulate cytokinesis [34,35]. Plk1 has recently been shown to also regulate microtubule plus-end dynamics [71] as well as midbody assembly [2]. Plk1 controls anaphase midzone microtubule elongation in part by regulating the activity of the chromokinesin KIF4, a PRC1 binding partner [70]. Interestingly, Aurora B also regulates microtubule dynamics through activating KIF4A at microtubule plus-ends [13,86,87]. In KIF4 knockdown HeLa cells, both the midzone length and cell length are increased with intercellular distances reaching up to 22 μm [71], a length also observed in NudC-deficient cells (Fig 6G). Whether NudC sits in the KIF4 pathway remains to be determined. Understanding how NudC phosphorylation by Plk1 and Aurora B are coordinated will further elucidate how these mitotic regulators control spindle dynamics and coordinate midbody assembly (Plk1 function) with abscission checkpoint (Aurora B function) to complete cytokinesis.
